# The complete mitochondrial genome of Western rainbowfish (*Melanotaenia australis* Castelnau, 1875)

**DOI:** 10.1080/23802359.2016.1168714

**Published:** 2016-04-19

**Authors:** Yuming Zhao, Zaizhong Chen, Jianzhong Gao, Lei Wang, Kai Lu

**Affiliations:** a Key Laboratory of Freshwater Fishery Germplasm Resources, Ministry of Agriculture, Shanghai, PR China;; b Shanghai Collaborative Innovation Center for Aquatic Animal Genetics and Breeding (ZF1206), Shanghai Ocean University, Shanghai, PR China

**Keywords:** *Melanotaenia australis*, mitogenome, rainbowfish

## Abstract

In this study, the mitochondrial genome of *Melanotaenia australis* Castelnau, 1875 (Atheriniformes:Melanotaeniidae) was sequenced for the first time. The assembled mitogenome consisting of 16,530 bp, includes 13 protein-coding genes, 22 transfer RNAs, two ribosomal RNAs genes and one putative control region. The overall base composition of *M. australis* is 27.79% for A, 29.66% for C, 15.90% for G, 26.66% for T and shows 92% identities to Lake Kutubu Rainbowfish, *Melanotaenia lacustris*. These data would provide useful molecular information for phylogenetic relationships within the family Melanotaeniidae species.

The *Melanotaenia australis* (Castelnau, 1875) is one of the most common and widespread freshwater fish in river systems of north-western Australia. *Melanotaenia australis* can grow to a length of around 10 cm, but are more common at 8 cm or less. Males are usually much larger and deeper bodied than females. Colouration generally consists of 1–2 broad, dark mid-lateral stripes and a series of narrow longitudinal stripes of orange-red corresponding with each scale row. The whole body has an overall sheen of iridescent purple or blue. Fins range from nearly colourless to deep red, or clear with red or green flecks. The second dorsal and anal fins have reddish orange rays with a yellow membrane and dark distal border (Allen 1978).

Samples of *M. australis* were wild captured from a river of the Kimberley region (north-western Australia), then the muscle was dissected and preserved in pure alcohol. The specimens were stored in Fish Specimens Museum in Shanghai Ocean University, the accession number is SHOU20150061001. Then their genomic DNA was extracted from muscle by using Sangon Mag-MK Animal Genomic DNA extraction kit (Sangon, Shanghai, China). The primers were designed according to the complete mitochondrial genome of *Melanotaenia boesemani* (KT380951) deposited in GenBank. PCR amplification and sequencing of the products were performed according to the method described by He et al. (2011) with slight modifications.

The complete mitochondrial genome of *M. australis* was 16,530 bp in size (GenBank accession no. KU529305), including 13 protein-coding genes, 22 transfer RNAs, two ribosomal RNAs genes and one putative control region. All protein-coding genes are encoded on H-strand with exception of protein-coding genes of ND6. All tRNA genes are encoded on H-strand with exception of *tRNA-Gln*, *tRNA-Ala*, *tRNA-Asn*, *tRNA-Cys*, *tRNA-Tyr*, *tRNA-Ser* (UGA), *tRNA-Glu* and *tRNA-Pro*. All the 13 mitochondrial protein-coding genes share the start codon ATG, except for *COI* (GTG start codon). The stop codon, TAA, is present in *COI*, *ND4L* and *ND6*; TAG is present in *ND1*, *ATP8*, *ND5* and *Cytb*; an incomplete stop codon ‘‘TA–’’ is found in *ND2*, *ATP6* and *COIII*; and ‘‘T– –’’ is found in *COII*, *ND3* and *ND4*. The longest one is *ND5* gene (1839 bp) in all protein-coding genes, whereas the shortest is *ATP8* gene (168 bp). The size of the 22 tRNA ranges from 67 bp to 74 bp. The two ribosomal RNA genes, 12S rRNA gene (945 bp) and 16S rRNA gene (1677 bp), are located between *tRNA-Phe* and *tRNA-Leu* (UAA) and separated by *tRNA-Val*.

The control region, which is 873 bp, is located between *tRNA-Pro* and *tRNA-Phe*. The overall base composition of *M. australis* is 27.79% for A, 29.66% for C, 15.90% for G, 26.66% for T, which is characteristic of mitochondrial genomes of other bony fish (Miya et al. 2003; Zhao et al. 2015a,b), and shows 92% identities to Lake Kutubu Rainbowfish, *M. lacustris* (Figure 1). We expect that the present result would elucidate the further phylogenetic approach among different species of rainbowfishes.

**Figure 1.**
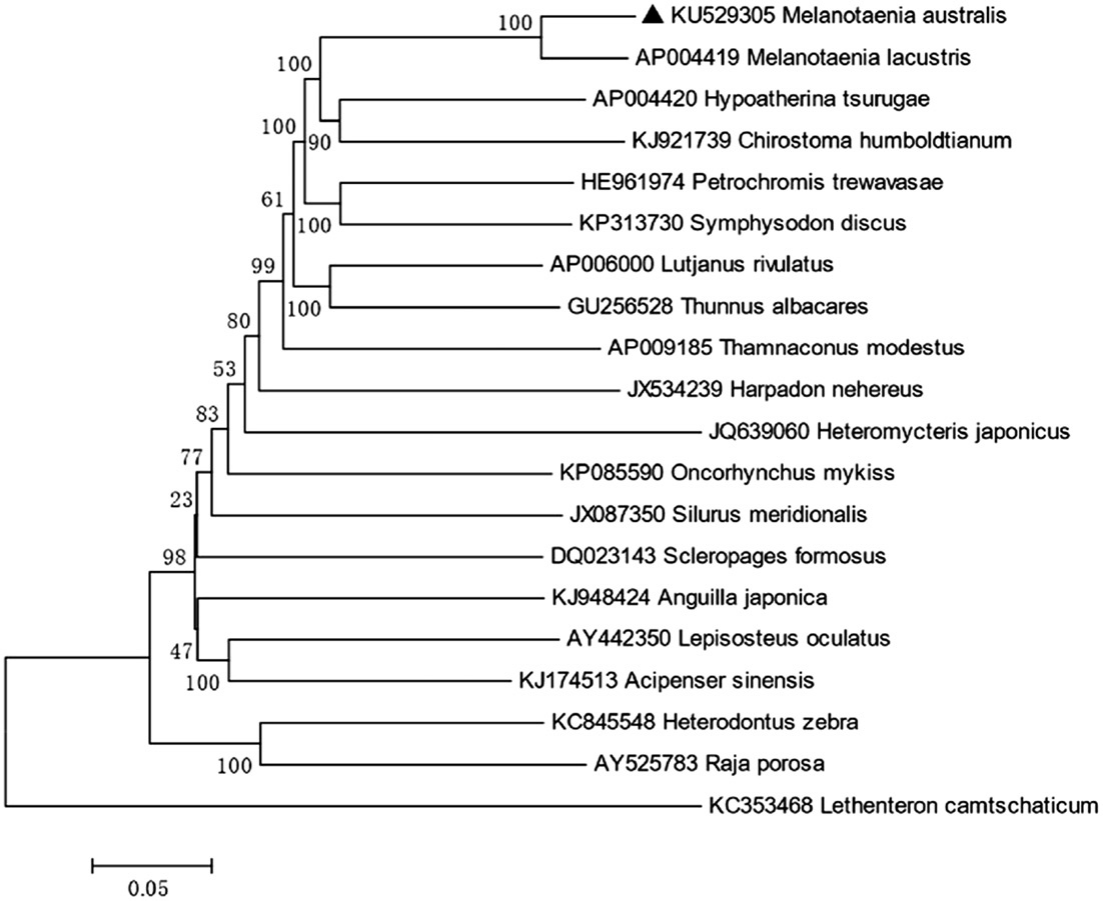
Neighbor-joining (NJ) tree of 20 species complete mitochondrial genome sequence. The phylogenetic relationships of *Melanotaenia australis* are close with *Melanotaenia.lacustris* using *Lethenteron camtschaticum* as an outgroup.
